# Lysine (protein) requirements of lactating sows

**DOI:** 10.1093/tas/txaa072

**Published:** 2020-05-25

**Authors:** Laura Greiner, Pariat Srichana, James L Usry, Casey Neill, Gary L Allee, Joseph Connor, Kevin J Touchette, Christopher D Knight

**Affiliations:** 1 Carthage Innovative Swine Solutions, LLC, Carthage, IL; 2 Department of Animal Science, University of Missouri-Columbia, Columbia, MO; 3 Ajinomoto Heartland LLC, Chicago, IL; 4 PIC, Hendersonville, TN; 5 Novus International, Inc., St. Charles, MO

**Keywords:** lactation, lysine, sow

## Abstract

Five experiments were conducted to evaluate the lysine (Lys) requirements of lactating sows. All diets were formulated to be isocaloric 3.46 Mcal ME/kg and met or exceeded National Research Council recommendations. In all studies, sow feed intake, body weight loss/gain, subsequent reproduction, and litter growth rate (LGR) were evaluated. The data were analyzed as randomized complete block design using generalized linear model in SAS with parity as a block. Two hundred and sixty-four primiparous sows (PIC Camborough 22) were randomly allotted to one of five lactation treatments (total Lys of 0.95%, 1.05%, 1.15%, 1.25%, and 1.35%) in Exp. 1 from August 2005 through October 2005. As daily total dietary Lys intake increased from 52.10 to 77.53 g, piglet ADG and daily litter gain linearly improved (*P* < 0.01). From February 2007 through April 2007, 336 multiparous sows (parity 4 and older, PIC Camborough 29) were randomly allotted to one of five lactation treatments (total Lys 0.85%, 0.95%, 1.05%, 1.15%, or 1.25%) in Exp. 2. As dietary total Lys increased from 0.85% to 1.25% of the diet, there were no significant differences in litter performance, such as ADG, daily litter gain, and the number of pigs weaned. Experiment 3 was conducted from October 2008 through January 2009. Two hundred and seventy-nine primiparous gilts (PIC Camborough 29) were randomly allotted to one of five lactation treatments (total Lys 1.14%, 1.25%, 1.35%, 1.46%, and 1.57%). Actual total Lys intakes increased from 56.74 to 77.12 g/d. Feeding total dietary Lys quadratically decreased (*P* < 0.01) weaning-to-estrus interval and increased percentage bred by 10 d (*P* = 0.02). In Exp. 4, 200 sows (parity 4 and older, PIC Camborough 29) were randomly allotted to one of five treatments (0.85%, 0.95%, 1.05%, 1.15%, or 1.25% total Lys) from January 2008 through March 2008. As dietary total Lys increased from 42.40 to 66.15 g/d, sow body weight and LGRs were not influenced by dietary total Lys intakes. In Exp. 5, 324 parity 3 sows (PIC Camborough 29) were randomly allotted to one of five treatments (0.77%, 0.92%, 1.08%, 1.23%, and 1.38% total Lys) from August 2009 through October 2009. As daily dietary total Lys intake increased from 39.44 to 67.32 g, the percentage of sows bred by 10 d increased (*P* = 0.02), as well as the LGR. A broken-line quadratic regression analysis demonstrated that the total Lys requirement for LGR for parity 1 females is calculated as 72.68 − [6.04 × (3.55 − LGR)] and for parity 3+ females as 92.03 − [11.9 × (4.24 − LGR)].

## INTRODUCTION

With lysine (Lys) being the first limiting amino acid, extensive studies have been conducted to investigate the requirement in lactating sows [National Research Council (NRC), 1998]. According to the NRC (2012) recommendations, estimates of amino acids for lactation can be predicated based on individual pig average daily gain (ADG) and litter size and hence milk production.

In assessing Lys requirements for lactating sows, two approaches could be made. Increasing crude protein (CP) levels to increase Lys concentration of the feed or directly increasing Lys and maintaining CP levels as constant. Strathe et al. (2017) demonstrated that feeding 850 g of standardized ileal digestibility (SID) CP per day optimized litter growth rate (LGR) across all parities by improving milk protein and yield. Additional studies have evaluated graded levels of dietary CP during lactation in an attempt to evaluate the response of sows to Lys intake (Stahly et al. 1990; Tritton et al. 1996; Touchette et al. 1998; Yang et al. 2000). King et al. (1993) suggested that higher dietary Lys is needed to minimize body nitrogen loss than for milk production. Tritton et al. (1996) demonstrated that feeding greater than 58 g/d total Lys as compared to feeding less than 37 g/d total Lys during the first parity increased subsequent litter size from 9.6 to 10.7 pigs born. Touchette et al. (1998) and Yang et al. (2000) demonstrated that increasing Lys intake above that required to maximize milk production decreased second litter size.

While many studies have focused on the total sow population in general, few studies have evaluated the requirements of the sow at different stages of physical development and parity. Huang et al. (2013) demonstrated that a reduction in first parity body weight loss is influenced by Lys level and an interactive effect of CP and Lys but not impacted singularly on increasing CP levels alone, while higher Lys levels may impact second parity females. It is recognized that the first parity gilt is a growing animal and, therefore, may require different levels of Lys compared to her older counterparts, thereby creating the hypothesis that the first parity gilt has a higher Lys requirement during lactation to support milk yield and also body development compared to the mature sow. Therefore, the objective of these studies was to determine the Lys requirement by increasing CP for lactating sows at different parities.

## MATERIALS AND METHODS

All studies were conducted in a commercial 6,000 sow farm located in Western Illinois of the United States. All animal care practices were conducted by following routine farm management procedures with the oversight of a herd veterinarian and Pork Quality Assurance (PQA) guidelines (National Pork Board, 2012); therefore, the experimental procedures were not submitted for approval by the Animal Care Committee.

### Experiment 1

Two hundred and sixty-four primiparous gilts (PIC Camborough 22, PIC USA, Hendersonville, TN) were farrowed from August 2005 through October 2005. The experimental lactation diets were formulated to contain different levels of total Lys (0.95%, 1.05%, 1.15%, 1.25%, and 1.35% respectively; Table 1). After farrowing, gilts were fed 1.8 kg on day 1, 2.7 kg on day 2, 3.6 kg on day 3, and then allowed ad libitum access to feed.

**Table 1.** Diet composition of sow lactation diets varying in dietary Lys for Exp. 1

	Percentage of total Lys				
Ingredient, %	0.95	1.05	1.15	1.25	1.35
Corn	66.65	62.90	59.15	55.65	52.10
Soybean meal, 48%	25.55	29.35	33.10	36.70	40.25
Choice white grease	4.00	4.00	4.00	4.00	4.00
Monocalcium phosphate, 21%	1.70	1.70	1.70	1.70	1.70
Limestone	1.25	1.20	1.20	1.15	1.15
Salt	0.50	0.50	0.50	0.50	0.50
VTM with phytase*	0.25	0.25	0.25	0.25	0.25
Choline chloride	0.10	0.10	0.10	0.10	0.10
Calculated composition					
ME (Mcal/kg)†	3.45	3.45	3.45	3.45	3.45
CP, %	19.70	20.20	20.69	22.17	23.64
Total Lys, %	0.95	1.05	1.15	1.25	1.35
SID Lys, %	0.82	0.92	1.01	1.10	1.19
Total Met + Cys‡, %	0.60	0.64	0.68	0.72	0.76
Total Thr, %	0.67	0.73	0.78	0.84	0.90
Total Trp, %	0.21	0.23	0.25	0.24	0.29
Total Val, %	0.84	0.91	0.92	1.05	1.12
Analyzed composition**					
CP, %	19.29	20.26	20.93	22.26	22.81
Total Lys, %	0.99	1.04	1.14	1.21	1.24
Total Met + Cys, %	0.64	0.66	0.71	0.72	0.72
Total Thr, %	0.70	0.72	0.79	0.83	0.84
Total Trp, %	0.23	0.24	0.24	0.25	0.26
Total Val, %	0.89	0.91	1.00	1.04	1.07

Ingredients are presented as percentage of inclusion in the diet and are reported on an “as-fed” basis.

*Vitamin and trace mineral (VTM) premix supplied per kilogram of diet: vitamin A, 11,000 IU; vitamin D_3_, 1,760 IU; vitamin E (dl alpha tocopheryl acetate), 66 IU; vitamin K (menadione activity), 3.96 mg; riboflavin, 8.25 mg; D-pantothenic acid, 29.70 mg; niacin, 49.5 mg; vitamin B_12_, 0.04 mg; D-biotin, 0.22 mg; folic acid, 1.65 mg; pyridoxine, 5.1 mg; Zn (ZnSO4), 170 mg; Cu (CuSO_4_), 20 mg; Fe (FeSO_4_), 170 mg; Mn (MnSO_4_), 50 mg; I (ethylenediamine dihydriodide), 0.35 mg; and Se (Na2SeO_3_), 0.30 mg. Phytase was provided as Optiphos (Huevepharma, Sofia, Bulgaria) and added 750 phytase units/kg diet.

†Calculated from NRC (1998) based on ME values of each ingredient.

‡M + C = methionine + cysteine.

**Diets were analyzed using HPLC at Ajinomoto Heartland Lab, Chicago, IL.

### Experiment 2

Three hundred and thirty-six multiparous sows (parity 4 and older, PIC Camborough 29, PIC USA, Hendersonville, TN) were farrowed from February 2007 through April 2007. Five dietary treatments were evaluated in this experiment (0.85%, 0.95%, 1.05%, 1.15%, or 1.25% total Lys, respectively; Table 2). After farrowing, sows were fed 1.8 kg on day 1, 2.7 kg on day 2, 3.6 kg on day 3, and then allowed ad libitum access to feed.

**Table 2.** Diet composition of sow lactation diets varying in dietary Lys for Exp. 2 and 4

	Percentage of total Lys				
Ingredient, %	0.85	0.95	1.05	1.15	1.25
Corn	70.20	66.54	62.90	59.26	55.65
Soybean meal, 48%	22.00	25.68	29.35	33.03	36.70
Choice White Grease	4.00	4.00	4.00	4.00	4.00
Monocalcium phosphate, 21%	1.70	1.68	1.67	1.66	1.65
Limestone	1.25	1.25	1.23	1.20	1.15
Salt	0.50	0.50	0.50	0.50	0.50
VTM with phytase*	0.25	0.25	0.25	0.25	0.25
Choline chloride	0.10	0.10	0.10	0.10	0.10
Calculated composition					
ME (Mcal/kg)†	3.45	3.45	3.45	3.45	3.45
CP, %	16.2	17.6	19.0	20.4	21.8
Total Lys, %	0.85	0.95	1.05	1.15	1.25
SID Lys, %	0.74	0.83	0.93	1.02	1.11
Total Met + Cys‡, %	0.57	0.61	0.65	0.68	0.72
Total Thr, %	0.61	0.67	0.73	0.79	0.84
Total Trp, %	0.19	0.21	0.23	0.25	0.27
Total Val, %	0.78	0.85	0.92	0.99	1.06
Analyzed composition**					
CP, %	16.10	17.16	18.70	20.67	22.56
Total Lys, %	0.87	0.89	1.01	1.08	1.21
Total Met + Cys, %	0.58	0.58	0.64	0.67	0.70
Total Thr, %	0.64	0.66	0.73	0.80	0.88
Total Trp, %	0.18	0.19	0.20	0.24	0.26
Total Val, %	0.80	0.81	0.89	0.97	0.99

Ingredients are presented as percentage of inclusion in the diet and are reported on an “as-fed” basis.

*Vitamin trace mineral (VTM) premix supplied per kilogram of diet: vitamin A, 13,201 IU; vitamin D_3_, 2,596 IU; vitamin E, 123.2 IU; vitamin K (menadione activity), 5.0 mg; riboflavin, 9.9 mg; D-pantothenic acid, 29.7 mg; niacin, 44.0 mg; vitamin B12, 0.04 mg; D-biotin, 0.85 mg; folic acid, 7.16 mg; thiamine, 8.8 mg; pyridoxine, 4.5 mg; chromium (chromium propionate), 0.40 mg; Zn (20% ZnSO4, 30% Zinc oxide, and 50% Mintrex Zn, Novus, St. Louis, MO), 150 mg; Cu (50% CuSo4 and 50% Mintrex Cu, Novus, St. Louis, MO), 15 mg; Fe (FeSO4), 100 mg; Mn (50% MnSO4 and 50% Mintrex Mn, Novus, St. Louis, MO), 50 mg; I (ethylenediamine dihydriodide), 0.4 mg; and Se (50% Na2Se and 50% organic Se)), 0.30 mg. Phytase was provided as Axtra (Danisco Animal Nutrition, Wiltshire, United Kingdom) and added 374 phytase units/kg diet.

†Calculated from NRC (1998) based on ME values of each ingredient.

‡M + C = methionine + cysteine.

**Diets were analyzed using HPLC at Ajinomoto Heartland Lab, Chicago, IL.

### Experiment 3

Two hundred and seventy-nine primiparous gilts (PIC Camborough 29, PIC USA, Hendersonville, TN) were farrowed from October 2008 through January 2009. Five dietary treatments were evaluated in this experiment (1.14%, 1.25%, 1.35%, 1.46%, and 1.57% total Lys, respectively; Table 3). After farrowing, gilts were fed 1.8 kg on day 1, 2.7 kg on day 2, 3.6 kg on day 3, and then were allowed 5.3 kg/d on day 4 after farrowing through the lactation phase.

**Table 3.** Diet composition of sow lactation diets varying in dietary Lys for Exp. 3

	Percentage of total Lys				
Ingredient, %	1.14	1.25	1.35	1.46	1.57
Corn	59.7	55.8	52.0	48.1	44.2
Soybean meal, 48%	32.5	36.4	40.3	44.2	48.1
Choice White Grease	4.0	4.0	4.0	4.0	4.0
Monocalcium phosphate, 21%	1.7	1.7	1.7	1.6	1.6
Limestone	1.3	1.3	1.3	1.2	1.2
Salt	0.50	0.50	0.50	0.50	0.50
L-threonine	0.0	0.00	0.01	0.01	0.01
VTM with phytase*	0.25	0.25	0.25	0.25	0.25
Choline chloride	0.10	0.10	0.10	0.10	0.10
Calculated composition					
ME (Mcal/kg)†	3.45	3.45	3.45	3.45	3.45
CP, %	20.5	22.0	23.5	25.5	26.6
Total Lys, %	1.14	1.25	1.35	1.46	1.57
SID Lys, %	1.00	1.10	1.20	1.30	1.40
Total Met + Cys‡, %	0.68	0.71	0.75	0.80	0.84
Total Thr, %	0.78	0.84	0.92	1.00	1.07
Total Trp, %	0.25	0.27	0.29	0.32	0.34
Total Val, %	1.07	1.13	1.21	1.29	1.36
Analyzed composition**					
CP, %	20.47	22.90	25.06	27.54	28.76
Total Lys, %	1.14	1.25	1.41	1.47	1.54
Total Met + Cys, %	0.63	0.64	0.72	0.81	0.84
Total Thr, %	0.77	0.90	0.97	1.04	1.10
Total Trp, %	0.23	0.27	0.30	0.33	0.34
Total Val, %	0.93	1.02	1.13	1.23	1.29

Ingredients are presented as percentage of inclusion in the diet and are reported on an “as-fed” basis.

*Vitamin and trace mineral (VTM) premix supplied per kilogram of diet: vitamin A, 11,000 IU; vitamin D_3_, 1,760 IU; vitamin E (dl alpha tocopheryl acetate), 66 IU; vitamin K (menadione activity), 3.96 mg; riboflavin, 8.25 mg; D-pantothenic acid, 29.70 mg; niacin, 49.5 mg; vitamin B_12_, 0.04 mg; D-biotin, 0.22 mg; folic acid, 1.65 mg; pyridoxine, 5.1 mg; Zn (ZnSO_4_), 170 mg; Cu (CuSO_4_), 20 mg; Fe (FeSO_4_), 170 mg; Mn (MnSO_4_), 50 mg; I (ethylenediamine dihydriodide), 0.35 mg; and Se (Na2SeO_3_), 0.30 mg. Phytase was provided as Optiphos (Huevepharma, Sofia, Bulgaria) and added 750 phytase units/kg diet.

†Calculated from NRC (1998) based on ME values of each ingredient.

‡M + C = methionine + cysteine.

**Diets were analyzed using HPLC at Ajinomoto Heartland Lab, Chicago, IL.

### Experiment 4

Two hundred sows (parity 4 and older, PIC Camborough 29, PIC USA, Hendersonville, TN) were farrowed from January 2008 through March 2008. Five dietary treatments were evaluated in this experiment (0.85%, 0.95%, 1.05%, 1.15%, or 1.25% total Lys, respectively; Table 2). After farrowing, sows were fed 1.8 kg on day 1, 2.7 kg on day 2, and 3.6 kg on day 3. After day 3, sows were allowed a maximum of 5.6 kg/d.

### Experiment 5

Three hundred and twenty-four parity 3 sows (PIC Camborough 29, PIC USA, Hendersonville, TN) farrowed during the time period of August 2009 through October 2009. Sows were fed one of five diets that contained different levels of total Lys (0.77%, 0.92%, 1.08%, 1.23%, 1.38%, respectively; Table 4). After farrowing, sows were fed 1.8 kg on day 1, 2.7 kg on day 2, and 3.6 kg on day 3. After day 3, sows were allowed a maximum of 5.6 kg/d.

**Table 4.** Diet composition of sow lactation diets varying in dietary Lys for Exp. 5

	Percentage of total Lys				
Ingredient, %	0.77	0.92	1.08	1.23	1.38
Corn	72.98	67.38	61.83	56.28	50.73
Soybean meal, 48%	19.20	24.80	30.35	35.90	41.45
Choice white grease	4.00	4.00	4.00	4.00	4.00
Monocalcium phosphate, 21%	1.70	1.70	1.70	1.70	1.70
Limestone	1.25	1.25	1.25	1.25	1.25
Salt	0.50	0.50	0.50	0.50	0.50
VTM with phytase*	0.28	0.28	0.28	0.28	0.28
Choline chloride	0.10	0.10	0.10	0.10	0.10
Calculated composition					
ME (Mcal/kg)†	3.45	3.45	3.45	3.45	3.45
CP, %	15.3	17.4	19.6	21.8	23.0
Total Lys, %	0.77	0.92	1.08	1.23	1.38
SID Lys, %	0.67	0.81	0.95	1.09	1.23
Total Met + Cys‡, %	0.53	0.59	0.65	0.71	0.77
Total Thr, %	0.57	0.65	0.74	0.82	0.91
Total Trp, %	0.17	0.20	0.24	0.27	0.29
Total Val, %	0.82	0.92	1.03	1.13	1.23
Analyzed composition**					
CP, %	16.16	19.09	20.11	22.56	24.30
Total Lys, %	0.87	0.96	1.09	1.17	1.27
Total Met + Cys, %	0.49	0.56	0.60	0.66	0.69
Total Thr, %	0.64	0.69	0.77	0.84	0.89
Total Trp, %	0.21	0.24	0.26	0.30	0.28
Total Val, %	0.77	0.86	0.93	1.03	1.08

Ingredients are presented as percentage of inclusion in the diet and are reported on an “as-fed” basis.

*Vitamin and trace mineral (VTM) premix supplied per kilogram of diet: vitamin A, 11,000 IU; vitamin D_3_, 1,760 IU; vitamin E (dl alpha tocopheryl acetate), 66 IU; vitamin K (menadione activity), 3.96 mg; riboflavin, 8.25 mg; D-pantothenic acid, 29.70 mg; niacin, 49.5 mg; vitamin B_12_, 0.04 mg; D-biotin, 0.22 mg; folic acid, 1.65 mg; pyridoxine, 5.1 mg; Zn (ZnSO_4_), 170 mg; Cu (CuSO_4_), 20 mg; Fe (FeSO_4_), 170 mg; Mn (MnSO_4_), 50 mg; I (ethylenediamine dihydriodide), 0.35 mg; and Se (Na2SeO_3_), 0.30 mg. Phytase was provided as Optiphos (Huevepharma, Sofia, Bulgaria) and added 750 phytase units/kg diet.

†Calculated from NRC (1998) based on ME values of each ingredient.

‡M + C = methionine + cysteine.

**Diets were analyzed using HPLC at Ajinomoto Heartland Lab, Chicago, IL.

### Lactation Feeding

Upon entering the farrowing unit and prior to farrowing, sows were fed 1.8 kg/d of the respective experimental, lactation diet. During lactation, sows were allowed either ad libitum access or a controlled amount of feed per day as defined within each experiment. Feed was delivered to each sow through the automated Howema Feed System (Big Dutchman, Vechta, Germany). In all studies, the low-Lys and high-Lys diets were manufactured at a local feed mill and delivered to the facility. The feed system on site blended the intermediate diets by delivering a set percentage of each diet into a mixing hopper and recording the actual weight of each diet as it was added to the hopper and then proceeded to mix the diet for 4 s prior to delivering each batch of feed to the corresponding sow. At the time of delivery, the system recorded the amount of feed delivered and tracked total lactation consumption per sow. Feed was delivered to each sow via a cable system and was held in a 6.8-kg plastic hopper (Automated Production Systems, Assumption, IL) attached to an InTak feeder (Automated Production Systems, Assumption, IL). Daily feed consumption was recorded, including feed refusals. Any feed not consumed remained in the hopper above the feed pan and the sow was able to remove the feed from the hopper the following day into the feed pan. Sows had ad libitum access to water throughout lactation.

During gestation and at weaning, sows received a common gestation diet. All lactation diets were isocaloric (3.45 Mcal/kg) and contained vitamins and minerals that exceeded NRC recommendations (NRC, 1998). Energy values for individual ingredients were calculated using the metabolizable energy (ME) values from the NRC (1998).

### Animal Husbandry

Sows were moved into the farrowing unit at 112 ± 2 d of gestation length and allocated to the experimental diets upon entry into the farrowing house and were fed the allotted treatments from the time of entry into the farrowing house until weaning. Sows were housed in conventional farrowing stalls in an environmentally regulated commercial farrow to wean facility (18–24 °C) with lights on from 0600 to 1500 h. Sows were housed in a standard farrowing stall with a total dimension of 1.5 × 2.1 m. Sows farrowed at 114 ± 3 d of gestation and piglets were cross-fostered within treatment within 24 h of birth. Tails of piglets were clipped and 200 mg of iron dextran was injected at 3 d of age. Male piglets were surgically castrated on day 3. Pigs were not offered creep feed during the study but did have access to water. In addition, rubber mats and heat lamps were provided as a source of supplemental heat to the piglets.

Pigs were weaned at approximately 19 ± 2 d of age. After weaning, sows were fed ad libitum a conventional gestation diet containing 3.17 Mcal ME/kg and 0.61% total Lys. Sows were checked daily for signs of estrus using a mature boar beginning day 3 after weaning.

### Sow and Litter Measurements

In all experiments, litters were negative for porcine epidemic diarrhea virus, transmissible gastroenteritis, porcine reproductive and respiratory syndrome virus (PRRS), and *Mycoplasma hyopneumoniae* stable. Sows were weighed at the time of entry into the farrowing house (Tru-Test, Mineral Wells, TX and J&H Automation, Gridley, IL) and again at the time of weaning. Sow 48-h postfarrow body weight was determined using the prediction equation: (0.9611) × [112-d prefarrow weight (kg)] − 9.288 (*R*^2^ = 0.94; R.D. Boyd, Hanor Company, Inc, Franklin, KY, unpublished data). In addition, piglet litter weights were recorded at 48 h of age and at weaning (Tru-Test, Mineral Wells, TX and J&H Automation, Gridley, IL). Piglets were cross-fostered within experimental treatment assignments within 24 h of birth to equalize litter number across treatments. Any mortalities and morbidities were recorded along with the piglet weights as the pigs were removed from trial. The removal weights and nursing days were calculated back into LGR (total litter wean weight − total starting litter weight + mortality weights)/[(number of pigs weaned × lactation length) + days mortalities nursed]. Estrus was recorded when sows stood to be mounted by a boar, and days from weaning to estrus were also recorded. In addition, the number of sows bred within 10 d of weaning was recorded. Weaning to mating interval, farrow to subsequent farrow interval and second litter number total born, born alive, stillborns, and mummies were recorded. For second parity litter characteristics, only sows mated within 21 d postweaning and farrowing as a result of first mating were used. Sows were removed from study due to feed valve not functioning correctly, mortality, prolonged illness, or milking fewer than seven piglets as this can cause estrus to occur during lactation and alter subsequent total born and weaning-to-estrus interval numbers.

### Data Quality Assurance

Litter counts were conducted every 3 d to verify that pigs were retained with their sow. Any litters in which the starting number plus/minus fostering and the removal of mortalities did not sum correctly were removed from the study. The feed system scale, sow body weight scale, and litter weight scale were calibrated every 6 mo by an outside vendor (J&H Automation, Gridley, IL). In addition, each time a body weight scale was used, a certified weight was placed onto the scale to verify the accuracy of the scale. Furthermore, the feed system scale weighing was validated with a certified weight at the beginning of each trial. Room temperature was recorded by the room ventilation controller, and a second thermometer located inside the room served as a validation of environment. Feed samples were collected from five individual feed hoppers for each dietary treatment for each study. Additional feed samples were also gathered from the feed bin at the farm for the high- and low-Lys diets and a sample was also retained at the mill prior to placement into the delivery truck. Data entry was completed and a second review of the data was conducted that included, but was not limited to, evaluation of minimum and maximum values for each parameter, standard deviation analysis, and random checking of specific litters for all parameter values entered.

### Diet Analysis

Diets were submitted to Ajinomoto Heartland, LLC (Chicago, IL) for amino acid and CP analysis (Association of Official Analytical Chemists, 1995). Analyzed amino acid values for each experiment are reported in Tables 1–4.

### Statistical Analysis

Data were analyzed by analysis of variance using MIXED procedures of SAS (Version 9.4, SAS Inst, Inc., Cary, NC) and reported as least square means. In all experiments, the fixed effect was treatment, and the sow was the experimental unit. The starting sow weight was used as a covariate in Exp. 3 and 5 and litter size standardization was used as a covariate in Exp. 4. Polynomial coefficients were used to determine linear and quadratic effects on increasing total Lys levels. A value of *P* < 0.05 was considered significant and a *P* < 0.10 was considered a trend. For each experiment, broken-line models were fitted to daily litter gain, subsequent total born, and weaning-to-estrus interval to further estimate total Lys requirements using NLMIXED in SAS [Robbins et al. (2006) and Pesti et al. (2009)]. Statistical models fitted to the data included a broken-line linear (BLL) ascending model and a broken-line quadratic (BLQ) ascending model. For the BLL ascending model: *yij = L*_BLL_*+ Ul* (*R*_BLL_*– Xi*) *+ bj + eij* for *Xi < R*_BLL_ and *yij = L*_*BLL*_*+ bj + eij* for *Xi ≥ R*_*BLL*_. For the BLQ ascending model: *yij = L*_BLQ_*+ Uq* (*R*_BLQ_*– Xi*)2 *+ bj + eij* for *Xi < R*_BLQ_ and *yij = L*_BLQ_*+ bj + eij for Xi ≥ R*_BLQ_. In these equations, *yij* is the response of the sow in the block *j* assigned to treatment *i*, *Xi* is the SID Tryp level of the *i*th dietary treatment, and *L*_BLL_ and *L*_BLQ_ indicate the unknown maximum response to the dietary treatments to reach plateau using the BLL and BLQ models. *R*_BLL_ and *R*_BLQ_ are the unknown minimum levels of the SID Trp required to reach plateau using the the BLL and BLQ models. Furthermore, *bj* is the random blocking effect of the parameter associated with the *j*th block and *eij* is the random error associated with the sow in the *j*th block that received the *i*th treatment.

Statistical models were compared using maximum likelihood-based fit criteria (BIC; Milliken and Johnson, 2009). The best-fitting model was reported with a 95% CI.

## RESULTS

### Experiment 1

In this study, gilts nursed for an average of 19.6 d. Gilts gained on average 5.2 kg of body weight with an average daily feed intake (ADFI) of 5.65 kg/d. Gilts started the trial with an average of 9.5 pigs per gilt and weaned 9.2 pigs per gilt with a piglet ADG of 0.266 kg/d and a daily LGR of 2.46 kg/d.

As daily total dietary Lys increased from 52.1 to 77.5 g, there was significant linear improvement in piglet ADG (*P* < 0.01) and average daily litter gain (*P* < 0.01; Table 5). There were no linear differences in gilt ADFI, gilt body weight change, or reproductive parameters, including percentage of gilts bred within 10 d postwean, weaning-to-estrus interval, and subsequent total born (*P* > 0.10); however, there were significant differences in LGR and average daily pig gain between the 52.1 and 77.5 g total Lys per day, as well as feed intake differences between Lys levels. Feeding primiparous sows with a 1.35% total Lys diet had the lowest weaning-to-estrus interval of 7.3 d and highest subsequent total born of 12.4 pigs per gilt.

**Table 5.** Experiment 1: evaluation of dietary total Lys level on primiparous sows and litters

		Percentage of total Lys								
	Parameter	0.95	1.05	1.15	1.25	1.35	SEM	*P* value	Linear	Quadratic
Sow										
	Number of sows	53	52	53	54	52				
	ADFI, kg	5.48^a,c^	5.85^b^	5.77^b,c^	5.40^a^	5.74^a,b,c^	0.12	0.02	0.86	0.43
	Total Lys, g/d	52.10^a^	61.47^b^	66.35^c^	67.54^c^	77.53^d^	1.36	<0.01	<0.01	0.63
	Gilt body weight (48 h), kg	188.92	191.33	194.30	187.50	191.99	2.73	0.41	0.79	0.58
	Weight change, kg	5.31	7.01	4.51	3.90	5.28	1.65	0.72	0.54	0.84
	Bred by 10 d, %	89.36	91.11	89.58	82.35	95.65	4.58	0.32	0.79	0.30
	Weaning-to-estrus interval, d	7.19	7.09	7.08	8.51	6.33	0.68	0.21	0.88	0.27
	Subsequent total born, *n*	12.82	12.73	12.22	12.13	12.20	0.50	0.76	0.22	0.68
Litter										
	Number of pigs started/sow, *n*	9.50	9.40	9.37	9.58	9.58	0.07	0.10	0.13	0.09
	Number of pigs weaned/sow, *n*	9.10	9.20	9.06	9.17	9.31	0.10	0.47	0.23	0.38
	Litter start weight, kg	15.89	15.88	15.96	16.29	16.41	0.32	0.65	0.15	0.66
	Litter wean weight, kg	56.28^a^	57.33^a^	57.71^a^	59.95^a^	62.58^b^	1.37	0.01	<0.01	0.32
	Piglet ADG, kg	0.253	0.262	0.266	0.270	0.279	0.006	0.06	<0.01	0.99
	Daily litter ADG, kg	2.32^a^	2.42^a,b^	2.44^b,c^	2.51^c,d^	2.61^e^	0.06	0.02	<0.01	0.77

Treatments with different superscripts denote statistical differences in means.

### Experiment 2

Sows nursed for an average of 19.8 d. Sows gained on average 11.5 kg of body weight with an ADFI of 7.42 kg/d. Sow weaning-to-estrus interval was 4.9 d. Sows started the trial with an average of 11.0 pigs per sow and weaned 10.0 pigs per sow with a piglet ADG of 0.265 kg/d and a daily LGR of 2.74 kg/d. Fifteen sows did not complete the lactation period of the study.

As dietary total Lys increased from 0.85% to 1.25% of the diet, there were no significant differences in litter performance, such as ADG, daily litter gain, and number of pigs weaned (Table 6; *P* > 0.10). Total dietary Lys consumed per day was linearly increased (*P* < 0.01) from 62.5 to 88.0 g, respectively. Furthermore, there were no significant differences on older sow weight gain, weaning-to-estrus interval, or subsequent total born as dietary total Lys increased (*P* > 0.10).

**Table 6.** Experiment 2: evaluation of total Lys on older parity (P4+) sows and litter performance

		Percentage of total Lys								
	Parameter	0.85	0.95	1.05	1.15	1.25	SEM	*P* value	Linear	Quadratic
Sow										
	Number of sows	65	64	66	63	63				
	ADFI, kg	7.48	7.51	7.40	7.68	7.03	0.20	0.22	0.25	0.20
	Total Lys intake, g/d	62.53^a^	70.86^b^	77.62^c^	88.53^d^	88.01^d^	2.25	<0.01	<0.01	0.09
	Sow 48 wt, kg	239.57	247.30	247.62	242.22	242.98	4.28	0.59	0.90	0.21
	Weight change, kg	15.26	10.17	12.19	12.67	6.72	2.96	0.32	0.11	0.76
	Bred by 10 d, %	100.00	98.39	96.83	100.00	98.28	1.50	0.49	0.69	0.41
	Weaning-to-estrus interval, d	4.82	5.13	5.07	4.74	4.85	0.22	0.61	0.61	0.40
	Subsequent total born, *n*	12.88	12.47	13.16	12.58	13.30	0.50	0.68	0.53	0.59
Litter										
	Number of pigs started/sow, *n*	10.83	11.25	11.02	10.98	10.89	0.13	0.18	0.71	0.09
	Number of pigs weaned/sow, *n*	10.03	10.17	10.12	10.0	9.90	0.17	0.85	0.42	0.59
	Litter start wt, kg	18.59	19.58	18.93	19.03	19.20	0.60	0.82	0.72	0.69
	Litter end wt, kg	66.04	66.53	66.55	66.65	65.40	1.72	0.98	0.83	0.59
	Piglet ADG, kg	0.267	0.262	0.268	0.266	0.264	0.005	0.89	0.82	0.91
	Daily litter ADG, kg	2.75	2.75	2.76	2.75	2.69	0.07	0.96	0.59	0.58

Treatments with different superscripts denote statistical differences in means

### Experiment 3

In Exp. 3, the gilts nursed for an average of 19.8 d. Ten animals were removed from study. Gilts lost on average 5.1 kg of body weight with an ADFI of 4.91 kg/d. Gilt weaning-to-estrus interval was 4.9 d. Gilts started the trial with an average of 10.6 pigs/ per sow and weaned 10.2 pigs per gilt with a piglet ADG of 0.239 kg/d and a daily LGR of 2.39 kg/d.

Actual total Lys intakes increased from 56.7 to 77.1 g/d as dietary Lys levels increased, respectively (Table 7; *P* < 0.01). There was no effect of dietary total Lys on ADFI (*P* > 0.10). Feeding increasing levels of total dietary Lys (56.7 to 70.2 g/d) quadratically decreased (*P* < 0.01) weaning-to-estrus interval and then weaning-to-estrus interval increased from 3.44 to 5.15 d when total dietary Lys increased from 70.2 to 77.1 g/d. As total dietary Lys increased from 56.7 to 70.2 g/d, the percentage bred by 10 d (*P* = 0.02; quadratic) increased; however, the percentage bred decreased as dietary Lys increased to 77.1 g/d. Piglet ADG also tended to be influenced quadratically (*P* = 0.08) when gilts were fed increasing levels of total Lys, respectively, with the greatest ADG occurring at 68.0 g of total Lys per day.

**Table 7.** Experiment 3: evaluation of total Lys on primiparous sows and litter performance

		Percentage of total Lys								
	Parameter	1.14	1.25	1.35	1.46	1.57	SEM	*P* value	Linear	Quadratic
Sow										
	Number of sows	57	55	52	55	51				
	ADFI, kg	4.96	4.84	5.02	4.81	4.92	0.09	0.38	0.69	0.83
	Total Lys intake, g/d	56.74^a^	60.22^a^	68.00^b^	70.24^b^	77.12^c^	1.16	<0.01	<0.01	0.78
	Gilt 48 h wt, kg	170.57	171.64	176.21	176.52	176.49	2.87	0.35	0.06	0.54
	Weight change, kg	−5.83	−4.93	−2.53	−3.30	−3.56	1.94	0.75	0.33	0.47
	Bred by 10 d, %	87.93	100.0	97.4	100.0	91.90	4.20	0.09	0.39	0.02
	Weaning-to-estrus interval, d	6.66^a^	4.71^b^	4.35^b^	3.44^b^	5.15^a,b^	0.62	0.03	0.05	<0.01
	Subsequent total born, *n*	11.29	12.84	12.40	12.74	12.67	0.56	0.23	0.12	0.23
Litter										
	Number of pigs started/sow, *n*	10.61	10.64	10.50	10.54	10.71	0.13	0.80	0.83	0.32
	Number of pigs weaned/sow, *n*	10.18	10.33	10.06	10.09	10.18	0.15	0.73	0.61	0.76
	Litter start wt, kg	17.47	19.98	17.23	17.68	17.43	0.45	0.80	0.79	0.84
	Litter wean wt, kg	58.83	60.51	60.68	59.14	60.63	1.36	0.76	0.59	0.67
	Piglet ADG, kg	0.235	0.238	0.245	0.240	0.236	0.004	0.43	0.78	0.08
	Daily litter ADG, kg	2.34	2.41	2.43	2.38	2.37	0.05	0.75	0.88	0.24

Treatments with different superscripts denote statistical differences in means.

### Experiment 4

One hundred and ninety sows completed the study. The average days nursed in the study were 19.0 d. Sows gained on average 2.9 kg of body weight with an ADFI of 5.31 kg/d. Sow weaning-to-estrus interval was 5.3 d. Sows started the trial with an average of 10.6 pigs per sow and weaned 9.2 pigs per sow with a piglet ADG of 0.224 kg/d and a daily LGR of 2.18 kg/d.

As dietary total Lys increased from 42.4 to 66.2 g/d, sow body weight and ADFI did not change (Table 8; *P* > 0.10). There was a tendency for the weaning-to-estrus interval to quadratically (*P* = 0.10) increase as dietary Lys intake increased from 42.4 to 52.4 g/d and then decrease as total Lys intake increased to 66.2 g/d. However, the percentage of sows bred by 10 d after weaning was not influenced with the trial average being 99.19% (*P* > 0.10). Additionally, the ADG for the piglets and LGRs were not influenced by dietary total Lys intakes up to 66.2 g/d (*P* > 0.10).

**Table 8.** Experiment 4: evaluation of total Lys on older parity (parity 4+) sows and litter performance

		Percentage of total Lys								
	Parameter	0.85	0.95	1.05	1.15	1.25	SEM	*P* value	Linear	Quadratic
Sow										
	Number of sows	38	36	35	40	40				
	ADFI, kg	5.40	5.25	5.16	5.07	5.33	0.17	0.47	0.59	0.13
	Total Lys intake, g/d	42.40^a^	47.41^a,b^	52.44^b^	57.18^b,c^	66.15^c^	1.73	<0.01	<0.01	0.13
	Sow 48 h wt, kg	257.87	268.49	260.04	272.83	263.62	5.46	0.24	0.34	0.35
	Weight change, kg	5.31	3.92	5.59	0.52	4.03	4.24	0.81	0.56	0.80
	Bred by 10 d, %	99.91	100.00	97.20	99.62	99.20	1.57	0.48	0.63	0.35
	Weaning-to-estrus interval, d	5.20	5.44	5.86	5.40	5.17	0.38	0.49	0.91	0.10
	Subsequent total born, *n*	10.27	10.70	11.03	10.87	10.12	0.89	0.88	0.96	0.30
Litter										
	Number of pigs started/sow, n*	10.45^a^	10.11^a^	10.60^a,b^	10.68^a,b^	10.93^b^	0.19	0.04	0.01	0.28
	Number of pigs weaned/sow, *n*	8.87	8.33	8.58	8.96	8.64	0.28	0.26	0.79	0.49
	Litter start wt, kg	17.15	17.06	15.96	16.00	16.44	0.83	0.55	0.22	0.36
	Litter wean wt, kg	50.44	50.40	47.73	52.01	51.02	2.82	0.72	0.69	0.54
	Piglet ADG, kg	0.220	0.231	0.219	0.231	0.233	0.01	0.65	0.33	0.84
	Daily litter ADG, kg	2.07	2.13	2.06	2.20	2.19	0.13	0.80	0.32	0.84

Treatments with different superscripts denote statistical differences in means.

*Starting pig number was used as a covariate in the analysis.

### Experiment 5

Three hundred and eight sows completed the study. The average days sows lactated for this study were 20.9 d. Sows lost on average 11.7 kg of body weight with an ADFI of 5.36 kg/d. Sow weaning-to-estrus interval was 5.03 d. Sows started the trial with an average of 12.0 pigs per sow and weaned 10.9 pigs per sow with a piglet ADG of 0.258 kg/d and a daily LGR of 2.81 kg/d.

There was no effect of diet on ADFI with sows averaging 5.4 kg/d (Table 9) as daily dietary total Lys intake increased from 39.4 to 67.3 g (*P* > 0.10). Increasing dietary Lys linearly increased (*P* = 0.03) the percentage of sows bred by 10 d and linearly decreased (*P* = 0.02) weaning-to-estrus interval. Subsequent total born was not changed with increasing dietary Lys intake with an average of 13.7 total born per sow (*P* > 0.10). In addition, the number of pigs weaned per sow and litter wean weight was significantly (*P* = 0.05) improved linearly as Lys levels increased. The average daily LGR was also linearly improved as dietary Lys intake increased (*P* < 0.01).

**Table 9.** Experiment 5: evaluation of total Lys on parity 3 sows and litter performance

		Percentage of total Lys								
	Parameter	0.77	0.92	1.08	1.23	1.38	SEM	*P* value	Linear	Quadratic
Sow										
	Number of sows	65	65	59	57	62				
	ADFI, kg	5.26	5.40	5.45	5.40	5.30	0.05	0.01	0.59	<0.01
	Total Lys intake, g/d	39.44^a^	47.61^b^	54.89^c^	61.54^d^	67.32^e^	0.44	<0.01	<0.01	<0.01
	Sow 48 h wt, kg	228.34^a^	219.01^b^	224.67^a,b^	218.08^b^	220.56^a,b^	2.83	0.04	0.05	0.27
	Weight change, kg	−18.67^a^	−11.06^b^	−10.18^b,c^	−5.73^c^	−12.07^b^	1.86	<0.01	<0.01	<0.01
	Bred by 10 d, %	94.89	98.05	99.00	97.81	100.00	1.58	0.15	0.03	0.49
	Weaning-to-estrus interval, d	5.88	4.97	4.99	5.28	4.52	0.32	0.06	0.02	0.62
	Subsequent total born, *n*	13.69	13.69	13.90	14.12	12.91	0.44	0.33	0.40	0.14
Litter										
	Number of pigs started/sow, *n*	12.03	11.92	12.00	12.02	12.10	0.12	0.87	0.53	0.47
	Number of pigs weaned/sow, *n*	10.82	10.82	10.68	11.04	11.23	0.17	0.15	0.05	0.16
	Litter start wt, kg	22.94	23.31	22.96	22.83	23.38	0.58	0.95	0.83	0.79
	Litter wean wt, kg	73.66	76.25	75.16	76.90	79.33	1.63	0.12	0.02	0.67
	Piglet ADG, kg	0.241	0.258	0.278	0.256	0.259	0.15	0.46	0.43	0.18
	Daily litter ADG, kg	2.66^a^	2.80^a,b^	2.77^a,b^	2.87^b^	2.97^b^	0.06	<0.01	<0.01	0.86

Treatments with different superscripts denote statistical differences in means

### Broken-Line Regression Analysis

**Figure 1.**
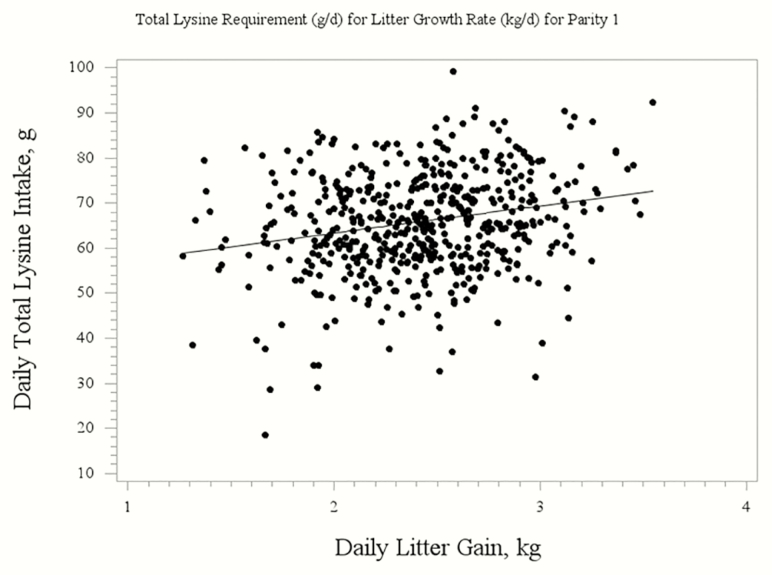
Broken-line quadratic regression analysis of total daily Lys intake requirement of the sow for optimal litter growth rate for first parity gilts. The regression equation is: 72.68 − [6.04 × (3.55 − LGR)] with a BIC of 4,093.

**Figure 2.**
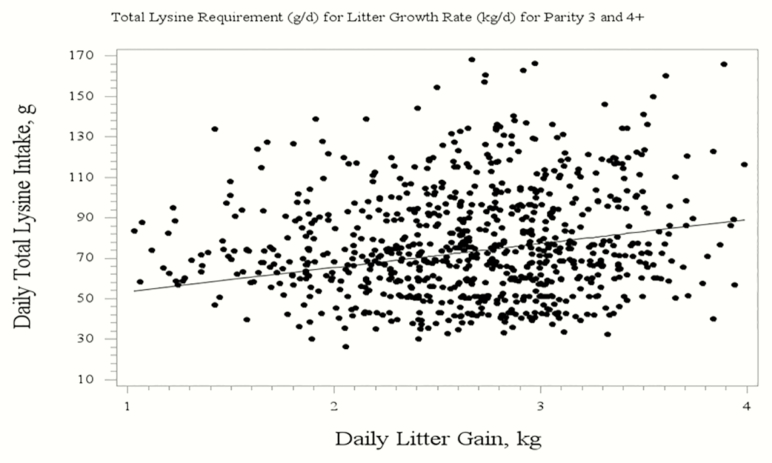
Broken-line quadratic regression analysis of total daily Lys intake requirement of the sow for optimal litter growth rate for mature females (parity 3 and 4+). The regression equation is: 92.03 − [11.90 × (4.24 − LGR)] with a BIC of 7,199.

**Figure 3.**
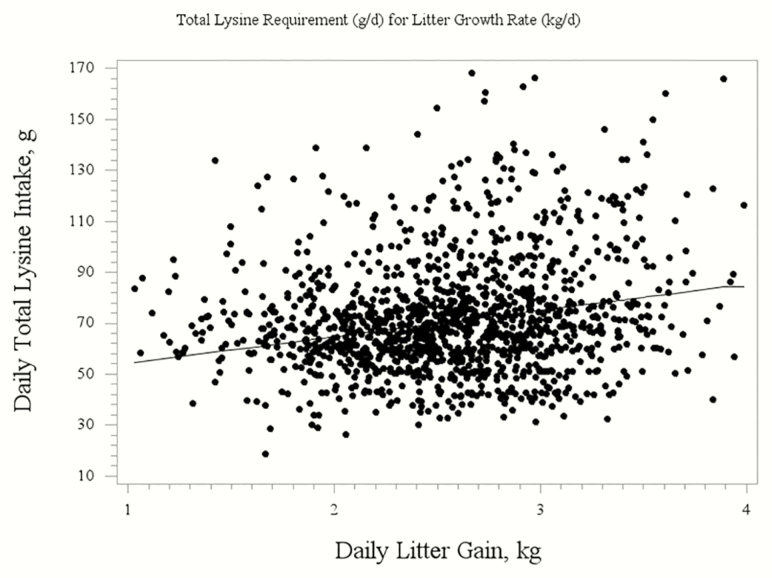
Broken-line quadratic regression analysis of total daily Lys intake requirement of the sow for optimal litter growth rate for all parities. The regression equation is: 84.14 − [10.31 × (3.55 − LGR)] with a BIC of 11,491.

**Figure 4.**
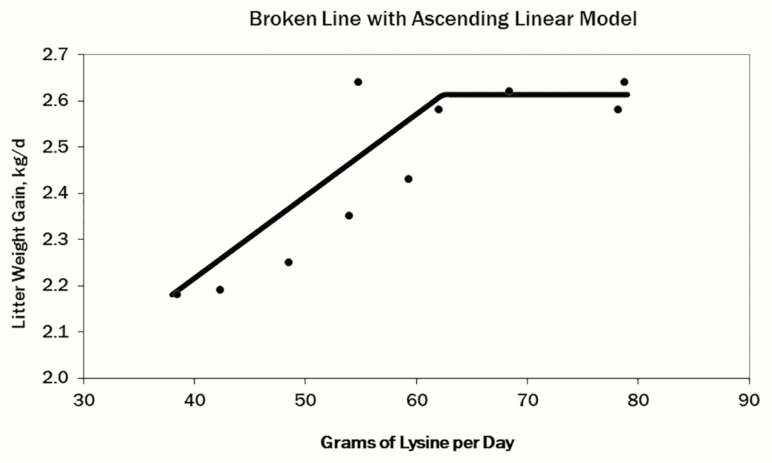
Broken-line with ascending linear model analysis of daily digestible Lys intake requirement (grams per day) of the sow for optimal litter growth rate (kilograms per day) for the older parity (P4+) sow. This graph combined the data from Exp. 2 and 4.

The evaluation of total Lys on LGR resulted in quadratic broken-line regression equations. For parity 1 sows, the regression to determine the daily total Lys requirement for optimal LGR was 72.68 − [6.04 × (3.55 − LGR)] (Fig. 1). For parity 3 and 4+ sows, the regression to determine the daily total Lys requirement for optimal LGR was 92.03 − [11.90 × (4.24 − LGR)] (Fig. 2). For an entire herd calculation, the regression to determine the daily total Lys requirement for optimal LGR was 84.14 − [10.31 × (3.55 − LGR)] (Fig. 3). A broken line with ascending linear model was used to combine the data from Exp. 2 and 4 for the P4+ lactating sow (Fig. 4) to demonstrate that the dietary digestible Lys requirement for optimal LGR was 63 g/d.

## DISCUSSION

In the five experiments, different levels of total Lys was provided, as well as differences in ad libitum intake and controlled feeding levels. However, in Exp. 2, the sows consumed beyond the expected levels and so allowances were made to minimize the effect of overconsuming the targeted grams per day levels in Exp. 4.

The studies were designed to define the Lys requirement for different parities of lactating sows. The diets in the studies did have increasing levels of CP as total Lys increased; however, Huang et al. (2013) demonstrated that CP level did not have a direct impact on sow performance. Crude protein along with high or low Lys levels did have an impact on reproductive performance. Furthermore, Greiner et al. (2018) demonstrated through a series of studies that reducing CP from 21% to 17% did not impact sow reproductive or litter performance when other amino acids, such as Trp, Thr, and Val, were kept in ratio to Lys above the sow’s requirements and Lys requirements were met. Therefore, the discussion will focus on the Lys levels and not CP levels as CP does not appear to impact reproductive or litter performance when all other nutrients meet or exceed the lactation requirements.

In Exp. 1, the increase in total Lys for first parity gilts resulted in a linear response of LGR to the increased total Lys intake; however, this response was not seen in Exp. 3. In Exp. 3, gilts had an improved weaning-to-estrus interval in relation to increased levels of total Lys intake. Upon further evaluation, lactating gilts in Exp. 1 had a positive body weight gain while in lactation and the gilts in Exp. 3 had a negative weight gain during a similar lactation period. The loss of response of litter ADG with increasing levels of total Lys intake in Exp. 3 was likely due to the protein mobilization of the lactating gilt to support milk production, thereby resulting in subsequent reproductive performance impact rather than immediate LGR (Dourmad et al., 1998). Furthermore, Mejia-Guadarrama et al. (2002) demonstrated that when sows are on a protein (Lys) restrictive diet, increasing Lys intake from 20 to 46 g resulted in improved ovulation rate but not weaning-to-estrus interval. In this study, gilts consumed between 52 and 77 g of total Lys, which was higher than the sow intake in Mejia-Guadarrama et al. (2002) study. The higher Lys intake while feed restricted may have resulted in a different outcome with weaning-to-estrus interval. Steverink et al. (1999) demonstrated that weaning-to-estrus interval is directly related to litter size; therefore, an improved weaning-to-estrus interval from Exp. 3 should correlate to improved ovulation rates and improved total born. While the total born in Exp. 3 was not significant, there were numerical improvements in total born (*P* = 0.12).

In Exp. 2, sows consumed higher than expected feed intakes resulting in high grams of total Lys consumption and elevated LGRs. A follow-up evaluation in Exp. 4 resulted in lower grams of total Lys intake per day and lower LGRs. Neither experiment resulted in a detectable difference in performance parameters associated with daily total Lys intake. However, it is important to note that the range of total Lys consumed per day in Exp. 2 was 62.5–88.0 g and the total Lys consumed per day in Exp. 4 was 42.4–66.0 g resulting in little overlap of grams of total Lys intake per day.

A factorial approach has been used to estimate amino acid requirements for zero N balance. Assuming 4 kg of milk is needed for each 1 kg of gain, a litter gaining 2.7 kg/d would require 10.8 kg of milk per day. Per the NRC (2012), a 200-kg lactating sow requires 2.46 g total Lys per day for maintenance, the milk contains 5.16% CP, and the milk has a Lys content of 7.01g/100 g of CP; thus, in this example, the sow is producing 39 g of total Lys per day in the milk. Assuming that the conversion of digestible N in milk protein is 70% (Everts and Dekker, 1994) and that dietary Lys is 85% digestible, 55.7 g digestible Lys (39/0.7) or 65.5 g total dietary Lys (55.7/0.85) would be required to satisfy the dietary requirements for milk Lys secretion. Thus, the lactating sow would require 67.96 g total Lys (65.5 + 2.46) per day to completely satisfy its needs for both milk production and body protein maintenance

A different factorial approach could be considered. Based on the example above, daily output of Lys in milk from a sow producing 10.8 kg of milk per day would be 43.0 g based upon milk containing 0.85% N (Whittemore, 2006) and Lys in sow milk being 7.5 g/16 g N. Assuming that the conversion of digestible N in milk protein is 70% (Everts and Dekker, 1994) and that dietary Lys is 85% digestible, 61.4 g digestible Lys (43.0/0.7) or 72.2 g total dietary Lys (61.4/0.85) would be required to satisfy the dietary requirements for milk Lys secretion. Thus, the lactating sow would require 74.66 g total Lys (72.2 + 2.46) per day to completely satisfy its needs for both milk production and body protein maintenance.

Other work conducted by Pettigrew et al. (1993) and followed by Boyd et al. (2000) formed an equation based on LGR to determine the total Lys requirement. The equation of [0.0266 × LGR(g/d)] − 7.549 would indicate that a sow nursing a litter growing at the rate of 2.7 kg/d would require 64.3 g of total Lys per day.

In the studies presented in this paper, regression equations to determine total Lys requirements based on LGR were developed using the data collected. The final equations were as follows: parity 1 gilts = 72.68 − [6.04 × (3.55 − LGR)] and parity 3+ sows = 92.03 − [11.90 × (4.24 – LGR)]. Based on the regression analysis conducted in these experiments, the mature female nursing a litter gaining 2.70 kg/d would require 73.70 g of total Lys per day. A younger female would require 67.6 g of total Lys per day to support a litter growing at the same rate of 2.70 kg/d. This analysis is further validated by evaluating the combined data for the parity 4+ sow, which requires approximately 63 g of SID Lys/d when LGR is 2.7 kg/d. Both values would be slightly higher than that estimate of Boyd et al. (1993) and slightly lower than that of the review conducted by Tokach et al (2019), which indicates that sows require 27 g of SID Lys per day per kilogram of LGR without sow body weight catabolism to contributing to the Lys requirement.

In these studies, it has been demonstrated that the older sow required similar total Lys to that of a growing, first parity gilt. Based on body weight and lactational parity, milk yield will be higher for the older sow by approximately 1.35 kg/d (Ngo et al., 2012). The increase in milk yield would increase total Lys requirements by 8.8 g/d for a sow. However, the younger gilt would not be mature for stature and, thereby, will require some protein for muscle and tissue development during the first lactation. Using the NRC (2012) estimates of SID Lys for maintenance for a lactating sow, a gilt at a weight of 190 kg would require 2.2 g of SID Lys (approximately 2.6 g total Lys) and a sow would require 2.8 g of SID Lys (approximately 3.3 g total Lys). Due to the increase in maintenance needs of 0.7 g/d and the milk yield increase of 12.4 g/d of a sow, a sow would require 9.5 g of total Lys more per day than a gilt. However, the younger gilt that is still growing will require Lys to go toward body growth during lactation. In Exp. 1, gilts gained approximately 300 g/d during lactation. Lysine content per 100 g of whole body weight gain is 7.10 g (NRC, 2012). The gain would result in 21.3 g of total Lys/d being needed for whole body weight gain. This will result in the gilt having a greater total Lys need over the sow by approximately 11.8 g/d, which was not what the data in these studies supported. One possible theory on why the sow requirement is similar to the gilt is that in some of the sow experiments, the sows gained weight and did not maintain or lose weight. Just as with the gilt, for each 100 g of whole body weight gain, the sow would need 7.10 g of total Lys, which would thereby make the total Lys needs equal between the two populations. In conclusion, the dietary Lys requirements for lactating sows of different parities are similar based on LGR with total Lys calculated for parity 1 females as 72.68 − [6.04 × (3.55 − LGR)] and for parity 3+ females as 92.03 − [11.9 × (4.24 − LGR)].
